# Bioinformatic identification and experiment validation reveal 6 hub genes, promising diagnostic and therapeutic targets for Alzheimer’s disease

**DOI:** 10.1186/s12920-023-01775-6

**Published:** 2024-01-02

**Authors:** Wenyuan Cao, Zhangge Ji, Shoulian Zhu, Mei Wang, Runming Sun

**Affiliations:** 1 Department of Neurology Second Ward, Zibo Municipal Hospital, No. 139, Huangong Road, Linzi District, Zibo City, 255400 Shandong Province China; 2 Department of Rehabilitation, Zibo Municipal Hospital, No. 139, Huangong Road, Linzi District, Zibo City, 255400 Shandong Province China

**Keywords:** Alzheimer disease, Gene expression profiling, Protein interaction maps, Computational biology

## Abstract

**Background:**

Alzheimer’s disease (AD) is a progressive neurodegenerative disease that can cause dementia. We aim to screen out the hub genes involved in AD based on microarray datasets.

**Methods:**

Gene expression profiles GSE5281 and GSE28146 were retrieved from Gene Expression Omnibus database to acquire differentially expressed genes (DEGs). Gene Ontology and pathway enrichment were conducted using DAVID online tool. The STRING database and Cytoscape tools were employed to analyze protein-protein interactions and identify hub genes. The predictive value of hub genes was assessed by principal component analysis and receiver operating characteristic curves. AD mice model was constructed, and histology was then observed by hematoxylin-eosin staining. Gene expression levels were finally determined by real-time quantitative PCR.

**Results:**

We obtained 197 overlapping DEGs from GSE5281 and GSE28146 datasets. After constructing protein-protein interaction network, three highly interconnected clusters were identified and 6 hub genes (*RBL1, BUB1, HDAC7, KAT5, SIRT2,* and *ITGB1*) were selected. The hub genes could be used as basis to predict AD. Histological abnormalities of brain were observed, suggesting successful AD model was constructed. Compared with the control group, the mRNA expression levels of *RBL1, BUB1, HDAC7, KAT5* and *SIRT2* were significantly increased, while the mRNA expression level of *ITGB1* was significantly decreased in AD groups.

**Conclusion:**

*RBL1, BUB1, HDAC7, KAT5, SIRT2* and *ITGB1* are promising gene signatures for diagnosis and therapy of AD.

**Supplementary Information:**

The online version contains supplementary material available at 10.1186/s12920-023-01775-6.

## Introduction

Alzheimer’s disease (AD), also known as senile dementia, is a common neurodegenerative disease that causes cognitive decline and dementia [1, 2]. During the past 20 years, the number of reported deaths induced by AD has been hugely increased [3]. There are over 50 million people globally with AD and the number of people with AD will exceed 100 million in 2050 [4]. The main cause of AD is the accumulation of beta-amyloid protein and misfolded microtubule-associated tau protein molecules in AD patients, damage nerves and other brain cells, which can lead to neuronal cell death and brain injury [5, 6]. Additionally, immune activation and astrocytic and glial cell-mediated neuroinflammation have been implicated in the pathogenesis of AD [7–9]. Only two medication classes—cholinesterase inhibitors and N-methylD-aspartate antagonists—are currently licensed for the treatment of AD [10]. Finding new therapeutic targets is urgently needed due to the limited treatments for AD.

Microarray analysis and gene sequencing have been widely applied to explore the potential biomarkers or therapeutic targets of AD. After using 3 microarray datasets to screen mitogen-related hub genes in peripheral blood mononuclear cells of AD patients, 53 differentially expressed genes (DEGs) of AD are identified, which may serve as possible biomarkers for the diagnosis of AD [11]. LncRNA RP11-59 J16.2 may be a molecular target for AD, according to microarray analysis of blood from AD patients and healthy controls to determine LncRNA and mRNA expression profiles [12]. After applying bioinformatics tools to analyze microarray profiling, nine blood-related differentially expressed miRNAs are identified, with potential as diagnostic biomarkers at an early stage [13]. Therefore, it is an important approach to explore and identify novel biomarkers and therapeutic targets associated with AD by screening for gene and molecular network changes related to the onset and progression of AD. However, the gene signature of AD has not been deeply explored.

In this study, we determined 6 hub genes of AD based on GSE5281 and GSE28146 datasets. And the expression of hub genes was further confirmed by constructing AD mouse model. The purpose of this investigation is to offer new knowledge on the molecular causes and gene biomarkers of AD.

## Materials and methods

### Expression profile datasets

The gene expression profiles we used were obtained from Gene Expression Omnibus (GEO) database https://www.ncbi.nih.gov/geo/). Datasets related to “Alzheimer’s disease” were retrieved, and two microarray datasets (GSE5281 and GSE28146) that met the criteria were selected.

### DEGs selection

The microarray data of the two datasets retrieved from the GEO database were conducted using the GEO2R (www.ncbi.nlm.nih.gov/geo/geo2r). DEGs were screened out according to a significance threshold with BH < 0.05 (BH: *P* value corrected by Benjamini-Hochberg multiple test) and |logFC| ≥ 1.5 (AD *vs*. Control). Heatmaps and volcano plots were plotted to visualize the identified DEGs. The samples of datasets were standardized and normalized using boxplots for data correction. Venn diagrams were created using Draw Venn Diagram tool (http://bioinformatics.psb.ugent.be/webtools/Venn/) to display the common DEGs between GSE5281 and GSE28146 datasets.

### Functional and pathway enrichment analysis

The Database for Annotation, Visualization and Integrated Discovery (DAVID) database was applied to carry out Gene Ontology (GO) and Kyoto Encyclopedia of Genes and Genomes (KEGG) enrichment analysis. Then, the GO and KEGG enrichment results were performed using R language. The most significantly enriched top 6 GO terms with the minimum adjusted *p*-values of each category and the top 8 significantly enriched pathways with the minimum adjusted p-value were selected for display, and enrichment analysis bar charts and bubble charts were created.

### Protein–protein interaction (PPI) network construction and hub gene identification

The STRING (https://www.string-db.org/) online database was employed to predict the PPI of DEGs. The significance level was set at a confidence interaction score of 0.4. Subsequently, the PPI network was displayed using Cytoscape software (www.cytoscape.org/). MCODE (Molecular Complex Detection) was employed to identify key modules from the PPI network of DEGs. The degree of each protein node was determined using the Cytoscape plugin CytoHubba Version 0.1 to filter out hub genes based on the connectivity.

### Hub gene analysis

A GO enrichment chord diagram was drawn to reveal the differences of hub genes in biological functions using R package ggplot2. The expression levels of hub genes in GSE28146 dataset were used as variables for principal component analysis (PCA). Two principal component variables, PC1 and PC2A were obtained after processing R language. The expression ridge plot was drawn using R language. Gene expression profile interactive analysis (http://gepia.cancer-pku.cn/) was applied to create a receiver operating characteristic (ROC) curve to assess the diagnostic accuracy of the hub genes according to the area under the curve (AUC).

### AD mouse model construction

ICR male mice (SPF Biotechnology Co.,Ltd., Beijing, China) at the age of 6–8 weeks, weighing 19–21 g, were separated into control group and model group (6 mice per group). After 1 week of adaptive feeding, the mice were intraperitoneally injected with 0.1 mL scopolamine at a daily dose of 1.5 mg/kg for 15 d to establish the model of AD. Mice were euthanized by intraperitoneal injection of 0.3% sodium pentobarbital (30 mg/kg body weight) and the brain tissue was extracted for subsequent assay.

Each mouse received unlimited food and was housed in a pathogen-free environment. The ethics committee gave the approval to the methods for caring for and using animals, and all relevant institutional and governmental guidelines for the ethical use of animals were followed.

### Real-time fluorescence quantitative PCR (RT-qPCR)

Total RNA was extracted from brain tissues using the TRIZOL reagent (Invitrogen, Carlsbad, CA, USA). A UV spectrophotometer was employed to measure the absorbance values at 260 nm and 280 nm to calculate the concentration of total RNA diluted 20 times with RNA se-free water. High purity samples (OD 260/OD 280 ratio between 1.9 and 2.0) were suitable for the following studies. Reverse transcription to synthesize cDNA templates was performed using a PCR amplification instrument. RT-qPCR were conducted using an ABI7500 Quantitative PCR instrument (Applied Biosystems, Foster City, CA, USA) with the following reaction procedures: pre-denaturation at 95 °C for 30 s, denaturation at 95 °C for 10 s, annealing at 60 °C for 30 s, and 40 cycles. GAPDH was used as an internal reference. The obtained Ct values were analyzed using the 2^-ΔΔCt^ method. Each experiment was repeated three times. The primer sequences were listed in Table S1.

### Hematoxylin-eosin (HE) staining

The mouse brain tissue was fixed in a 4% paraformaldehyde solution. The next day, after washing away paraformaldehyde solution, and the tissue was dehydrated using a gradient of 50–70% - 80 - 90% - 95% ethanol. Then the tissue was placed in a mixture of 1:1 ethanol and xylene for 30 min, and then transferred to pure xylene for transparency. The tissue was embedded in a paraffin solution. The paraffin portions were in an oven at 60 °C and roasted for 2 h and dewaxed from xylene to water using routine xylene and ethanol treatments. Sections were stained with hematoxylin and eosin for 10 min and observed using an optical microscope.

### Statistical analysis

Data processing was conducted using GraphPad Prism 7.0, which were presented as mean ± standard deviation. Comparisons between two groups were conducted by t-test, and *P* < 0.05 showed a statistical significance.

## Results

### Microarray data and DEGs identification

**Fig. 1:**
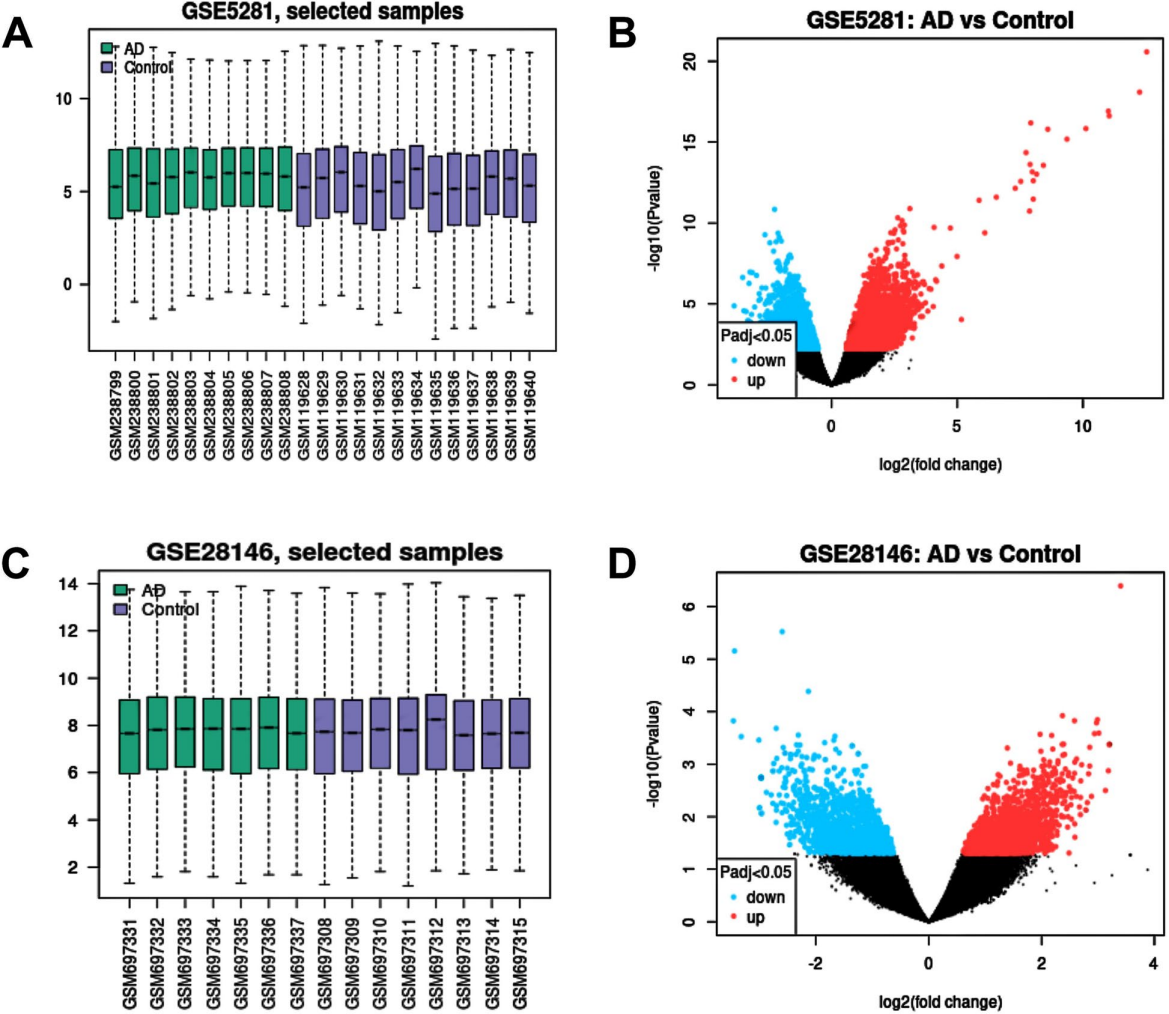
**a**, **c** Box plots of the data normalization results for the dataset samples. **b**, **d** Volcano plots with log2 Fold Change as the x-axis and -log10 (*p*-value) as the y-axis. Red dots represent up-regulated genes, and blue dots represent down-regulated genes

Two gene expression profile datasets were selected for this study, GSE5281 and GSE28146. After normalization and removal of batch effects, we selected total 23 samples in GSE5281 and 15 samples in GSE28146 for further analysis (Fig. 1a, c). The gene dataset GSE5281 consisted of 13 normal brain samples (Control) and 10 AD samples. Using GEO2R, 2780 DEGs were isolated in GSE5281, with 2198 up-regulated genes and 582 down-regulated genes. The GSE28146 dataset included 8 normal brain samples (Control) and 7 AD samples. There were 1123 DEGs were identified, among which 604 genes were up-regulated and 519 genes were down-regulated. Clustering analysis was performed on DEGs from both datasets and generated the volcano plots (Fig. 1b, d).

**Fig. 2:**
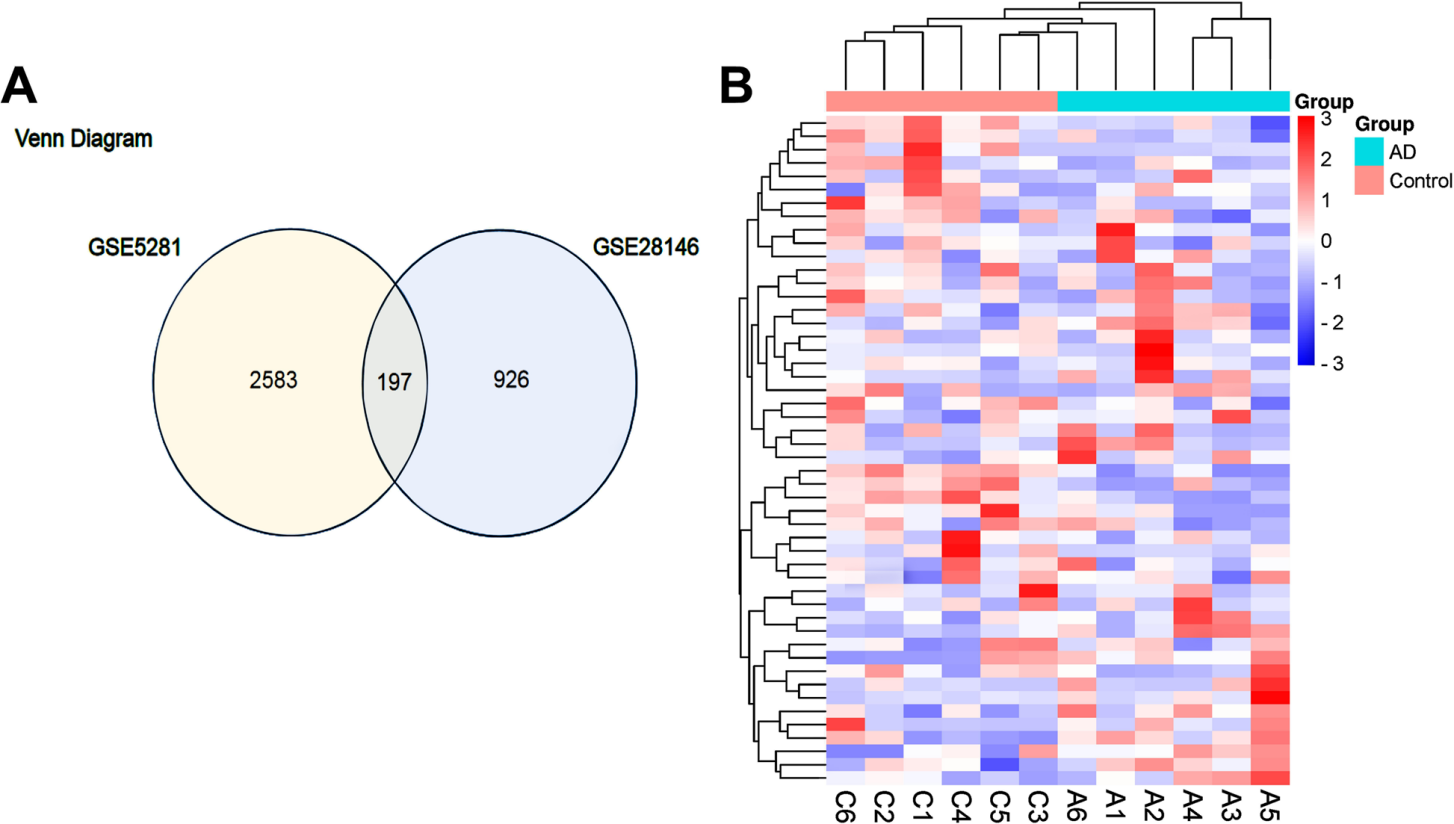
**a** Venn diagram of common differentially expressed genes (DEGs). The sum of the numbers of each circle represents the total number of DEGs, and the overlapping regions indicate the common DEGs between comparison groups. **b** Heatmap representation of the selected genes. Each column represents a sample and the horizontal axis represents genes. Red indicates high expression, and blue indicates low expression

A Venn diagram revealed that there were 197 common DEGs between the GSE5281 dataset and the GSE28146 dataset (Fig. 2a). Subsequently, the top 25 significantly up-regulated and down-regulated DEGs were selected, and a heatmap was created for visualization (Table 1) (Fig. 2b).

**Table 1:** Common differentially expressed gene of GSE5281 and GSE28146 datasets

Name	Description	log2FoldChange	pval	up/down
YME1L1	YME1 like 1 ATPase	2.97	1.63E-04	**up**
KMT2A	lysine methyltransferase 2A	2.69	4.30E-03	**up**
C10orf90	chromosome 10 open reading frame 90	2.62	7.98E-04	**up**
EGFR	epidermal growth factor receptor	2.49	4.91E-02	**up**
FARP2	FERM, ARH/RhoGEF and pleckstrin domain protein 2	2.44	3.46E-03	**up**
CMYA5	cardiomyopathy associated 5	2.38	2.07E-03	**up**
NSUN3	NOP2/Sun RNA methyltransferase family member 3	2.31	1.45E-02	**up**
MAGI1	membrane associated guanylate kinase, WW and PDZ domain containing 1	2.28	3.57E-03	**up**
LOC101927027	uncharacterized LOC101927027	2.25	4.95E-03	**up**
NPAS3	neuronal PAS domain protein 3	2.23	1.36E-02	**up**
SLC25A37	solute carrier family 25 member 37	2.22	4.05E-03	**up**
NFYA	nuclear transcription factor Y subunit alpha	2.21	8.77E-03	**up**
SESN2	sestrin 2	2.19	2.80E-03	**up**
PIK3C2A	phosphatidylinositol-4-phosphate 3-kinase catalytic subunit type 2 alpha	2.17	3.42E-02	**up**
PARD3B	par-3 family cell polarity regulator beta	2.16	5.22E-03	**up**
PAX8	paired box 8	2.15	3.87E-03	**up**
OR5J2	olfactory receptor family 5 subfamily J member 2	2.15	3.63E-03	**up**
IQSEC3	IQ motif and Sec7 domain 3	2.14	1.07E-03	**up**
EP400	E1A binding protein p400	2.13	3.60E-02	**up**
DOCK9	dedicator of cytokinesis 9	2.12	2.88E-02	**up**
MAP3K6	mitogen-activated protein kinase kinase kinase 6	2.08	1.21E-02	**up**
PHF19	PHD finger protein 19	2.06	8.62E-03	**up**
BUB1	BUB1 mitotic checkpoint serine/threonine kinase	2.05	1.77E-02	**up**
ELK4	ELK4, ETS transcription factor	2.04	5.34E-03	**up**
PRB1	proline rich protein BstNI subfamily 1	2.04	8.28E-04	**up**
EIF1AY	eukaryotic translation initiation factor 1A, Y-linked	−2.71	1.28E-02	**down**
MYH11	myosin heavy chain 11	−2.58	4.80E-04	**down**
MIR4683///FZD8	microRNA 4683///frizzled class receptor 8	−2.54	7.25E-03	**down**
COL11A1	collagen type XI alpha 1 chain	−2.46	2.52E-02	**down**
LOC100996385	uncharacterized LOC100996385	−2.45	7.87E-04	**down**
DUSP16	dual specificity phosphatase 16	−2.37	2.53E-03	**down**
FBXL17	F-box and leucine rich repeat protein 17	−2.37	1.40E-02	**down**
KIAA1841	KIAA1841	−2.33	7.73E-03	**down**
SLF2	SMC5-SMC6 complex localization factor 2	−2.28	4.31E-04	**down**
TMLHE	trimethyllysine hydroxylase, epsilon	−2.28	7.54E-03	**down**
VPS53	VPS53, GARP complex subunit	−2.25	8.13E-03	**down**
CDH7	cadherin 7	−2.14	2.08E-02	**down**
MYLIP	myosin regulatory light chain interacting protein	−2.13	9.11E-03	**down**
CAMSAP1	calmodulin regulated spectrin associated protein 1	−2.11	3.82E-03	**down**
SCFD1	sec1 family domain containing 1	−2.07	1.43E-02	**down**
CEP350	centrosomal protein 350	−2.06	1.12E-02	**down**
NMNAT2	nicotinamide nucleotide adenylyltransferase 2	−2.06	1.22E-02	**down**
DRP2	dystrophin related protein 2	−2.04	2.51E-02	**down**
TMF1	TATA element modulatory factor 1	−2.03	2.20E-02	**down**
CACNG4	calcium voltage-gated channel auxiliary subunit gamma 4	−2.03	6.73E-04	**down**
ARHGEF28	Rho guanine nucleotide exchange factor 28	−2.02	3.06E-02	**down**
FAM86B3P	family with sequence similarity 86, member A pseudogene	−2.02	2.45E-02	**down**
IKZF3	IKAROS family zinc finger 3	−2.00	2.24E-02	**down**
UBASH3B	ubiquitin associated and SH3 domain containing B	−1.97	1.34E-02	**down**
NELFCD	negative elongation factor complex member C/D	−1.97	1.85E-02	**down**

### Functional enrichment analysis of common DEGs

**Fig. 3:**
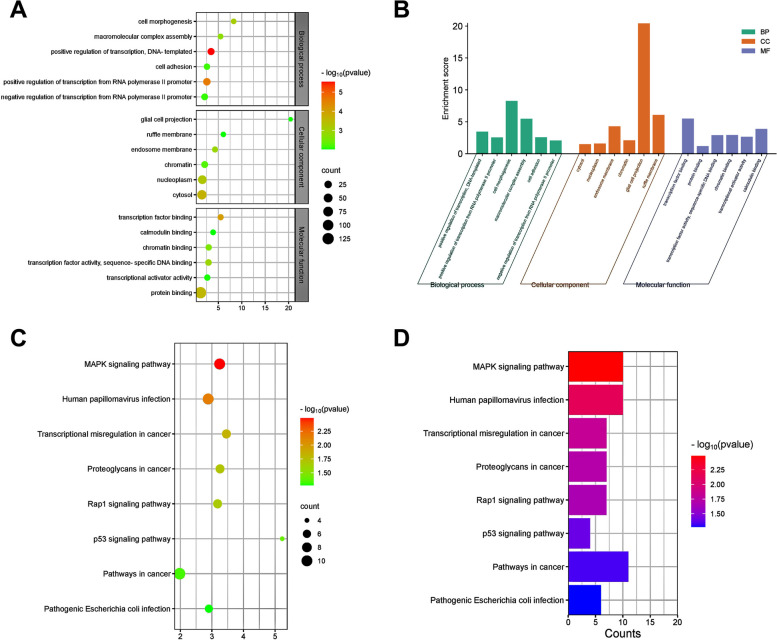
Enrichment analysis of the common DEGs. **A** Gene Ontology (GO) enrichment analysis bubble plot. The color intensity of nodes represents the adjusted *p*-value, and the node size indicates the number of genes. **B** GO enrichment analysis bar plot. The x-axis represents the GO terms, and the y-axis represents the -log10 (*p*-value) of enrichment of each term. **C** Kyoto Encyclopedia of Genes and Genomes (KEGG) bubble plot. D: KEGG bar plot

The molecular function (MF), biological process (BP), and cellular component (CC) categories grouped the results of the GO enrichment study. The GO enrichment analysis bubble plot and the GO enrichment analysis bar plot displayed the top 6 notably enriched GO terms (Fig. 3a, b). For the MF, the DEGs mainly enriched in “transcription factor binding”, “protein binding”, “transcription factor activity”, “chromatin binding”, “transcriptional activator activity”, “calmodulin binding”. For the BP, the DEGs mainly enriched in “positive regulation of transcription, DNA-templated”, “positive regulation of transcription from RNA polymerase II promoter”, “cell morphogenesis”, “macromolecular complex assembly”, “cell adhesion”, “negative regulation of transcription from RNA polymerase II promoter”. And for the CC, the DEGs mainly enriched in “cytosol”, “nucleoplasm”, “endosome membrane”, “chromatin”, “glial cell projection”, “ruffle membrane”.

Based on the KEGG enrichment analysis results of the DEGs, the top 8 enriched pathways were displayed in the KEGG pathway enrichment analysis bubble plot and the KEGG enrichment analysis bar plot, which mainly enriched in “MAPK signaling pathway”, “human papillomavirus infection”, “transcriptional mis regulation in cancer”, “proteoglycans in cancer”, “Rap1 signaling pathway”, “pathways in cancer”, etc. (Fig. 3c, d).

### PPI construction and key module analysis

Utilizing the STRING tool, a PPI network based on DEGs was constructed (Fig. S1A). Subsequently, MCODE (Molecular Complex Detection) was used to identify three highly interconnected clusters from the PPI network of DEGs, which potentially represented functional molecular complexes associated with AD. *RBL1, BUB1, HDAC7, KAT5, SIRT2* and *ITGB1* were selected as hub genes from these molecular (Fig. S1B).

### Analysis of hub genes

**Fig. 4:**
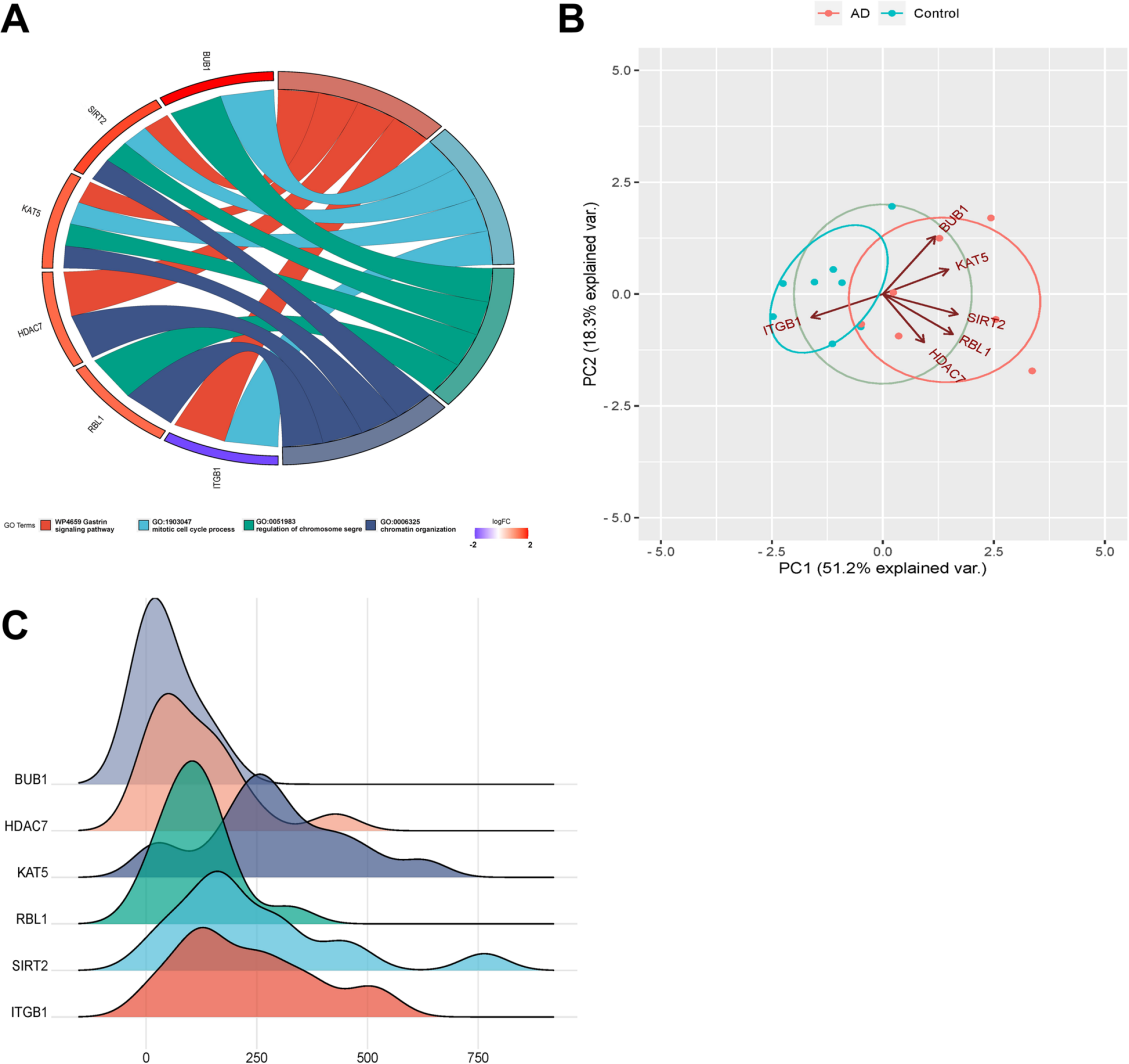
Hub gene analysis **a** GO pathway diagram, consisting of three parts: genes, LogFold Change (representing the fold change of genes for sorting and color-coding gene blocks), and other columns representing GO terms. Different connections between genes indicate their involvement in specific GO terms. **b** Principal component analysis (PCA) plot. The coordinates PC1 and PC2 represent the first and second principal components (i.e., latent variables explaining the differences). Points represent samples, and different colors represent different groups. **c** Gene ridge plot. The x-axis represents gene expression levels, and the shape of the ridges represents the distribution of data within each group, with the height indicating the number of samples corresponding to the gene expression level

We used R language to create a GO enrichment chord diagram, which revealed the differences of hub genes in biological functions and the relationship between protein and pathway of hub genes. The left side of the GO enrichment chord diagram was the hub gene sequenced according to Log Fold Change, and the right side was the GO term list. The hub gene with the significantly up-regulated differential fold was BUB1, and the hub gene with the significantly down-regulated differential fold was ITGB1. The biological process of the hub genes was mainly enriched in “gastrin signaling pathway”, “mitotic cell cycle process”, “regulation of chromosome” and “chromatin organization” (Fig. 4a).

PCA analysis revealed that hub genes were the key influencing factors of AD (Fig. 4b). The scatter plot (PC1 and PC2 as the horizontal and vertical coordinates) suggested the total variance explained rate of PC1 and PC2 was 69.5% and the samples had a good separation, further confirming the effectiveness of PC1 and PC2, which indicating that hub genes could distinguish between the AD samples from control samples. To visualize the expression level of hub genes in the original sample, we plotted the ridgeline plot using R language, showing the distribution of hub gene expression (Fig. 4c). These indicators could serve as a basis for distinguishing between control samples and AD samples.

**Fig. 5:**
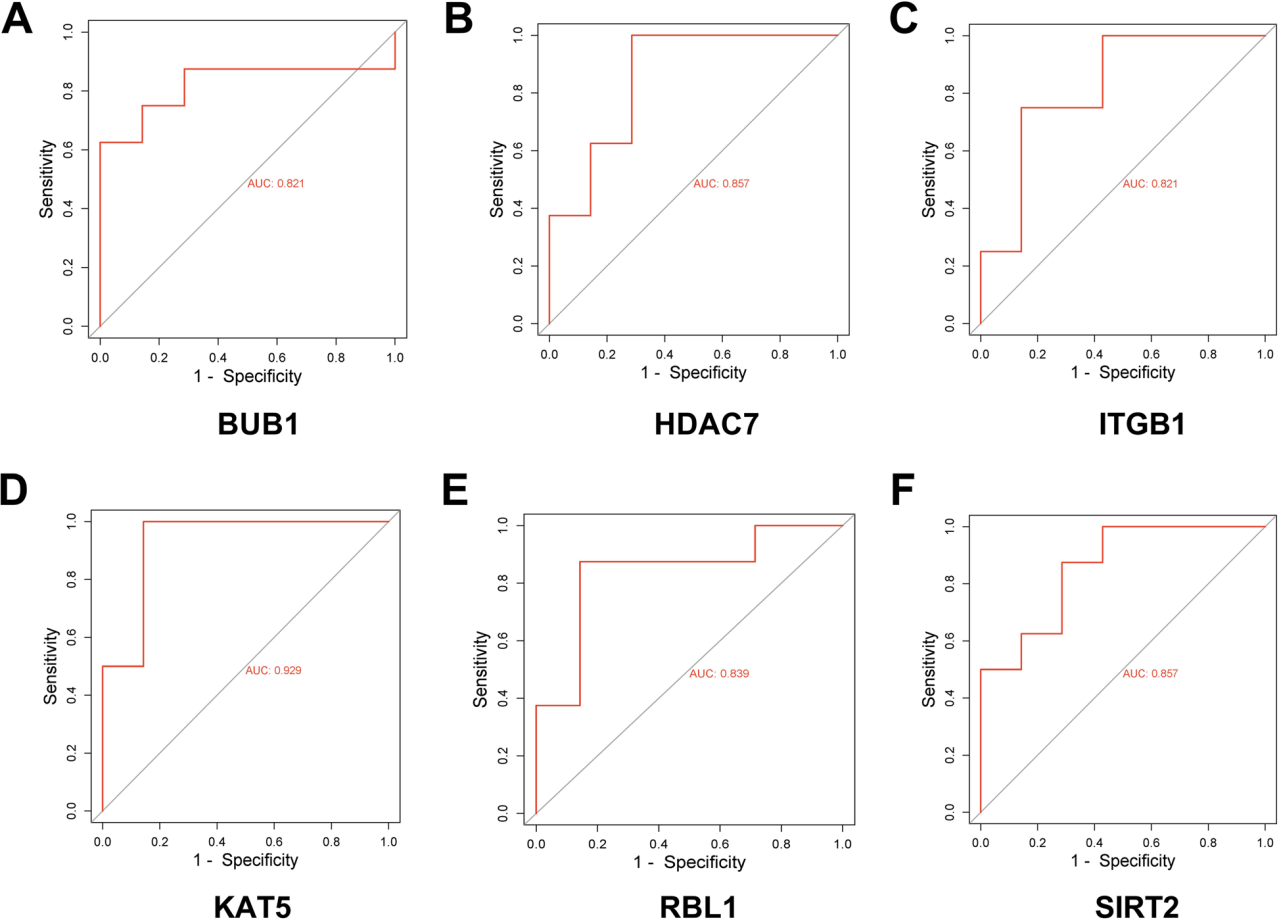
Receiver operating characteristic (ROC) curve plotted using the expression level of hub gene in GSE28146 dataset. **a**-**f** ROC analysis plots for genes *RBL1, BUB1, HDAC7, KAT5, SIRT2, and ITGB1*. The x-axis in ROC curve represents the false positive rate, the y-axis represents the true positive rate

**Fig. 6:**
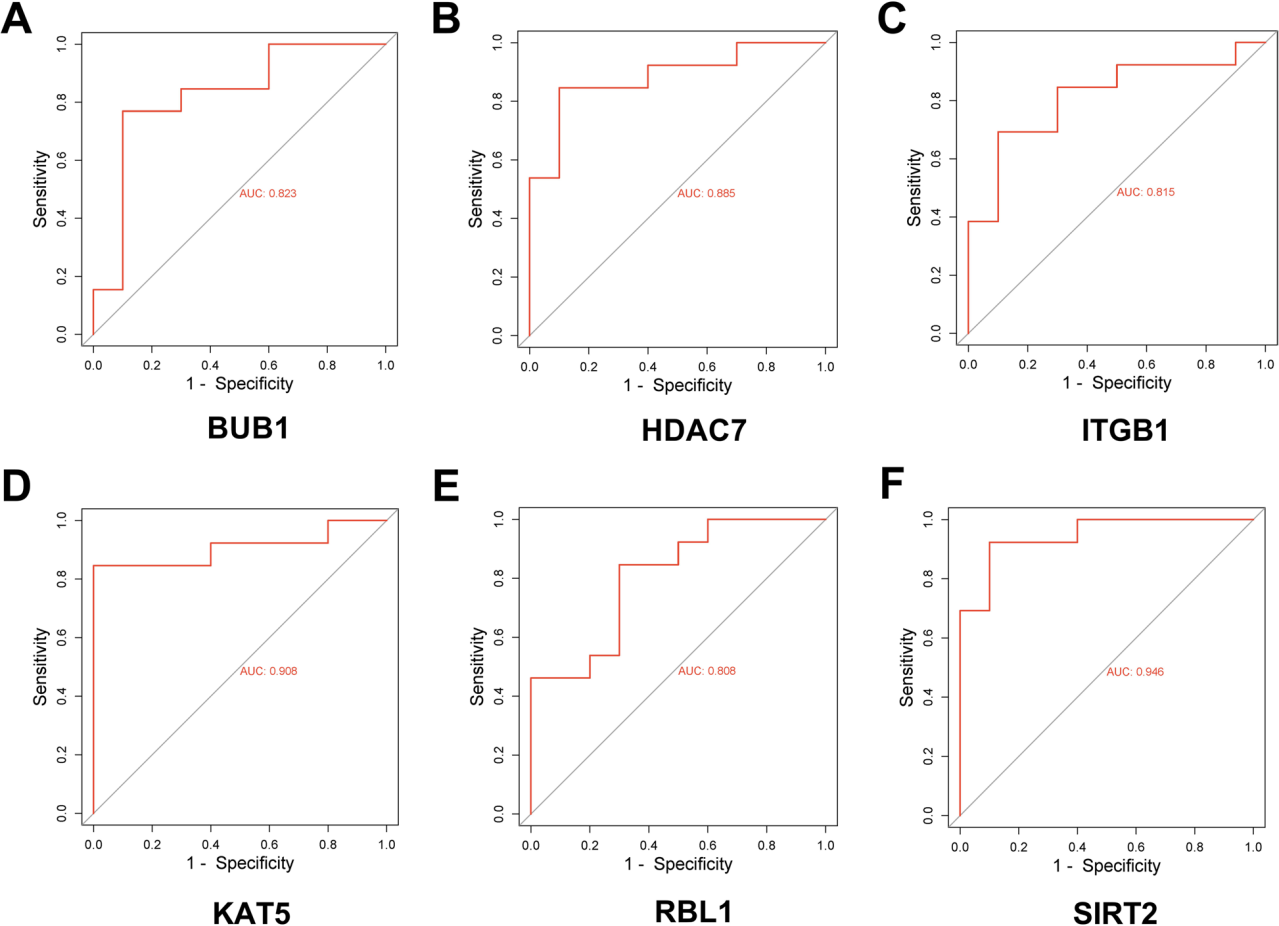
ROC curve plotted using the expression level of hub gene in GSE5281 dataset. **a**-**f** ROC analysis plots for genes *RBL1, BUB1, HDAC7, KAT5, SIRT2, and ITGB1*. The x-axis in ROC curve represents the false positive rate, the y-axis represents the true positive rate

The ROC curves of *RBL1, BUB1, HDAC7, KAT5, SIRT2*, and *ITGB1* were plotted using raw data from GSE28146 dataset, with the true positive rates of 83.9, 82.1, 85.7, 92.9, 85.7, and 82.15%, respectively (Fig. 5). The true positive rates of ROC curves plotted using raw data from GSE5281 dataset of hub genes were 82.3, 88.5, 81.5, 90.8, 80.8, and 94.6%, respectively, which showed a distinguishing capacity of hub genes expression levels between AD samples and healthy controls (Fig. 6).

### HE staining

**Fig. 7:**
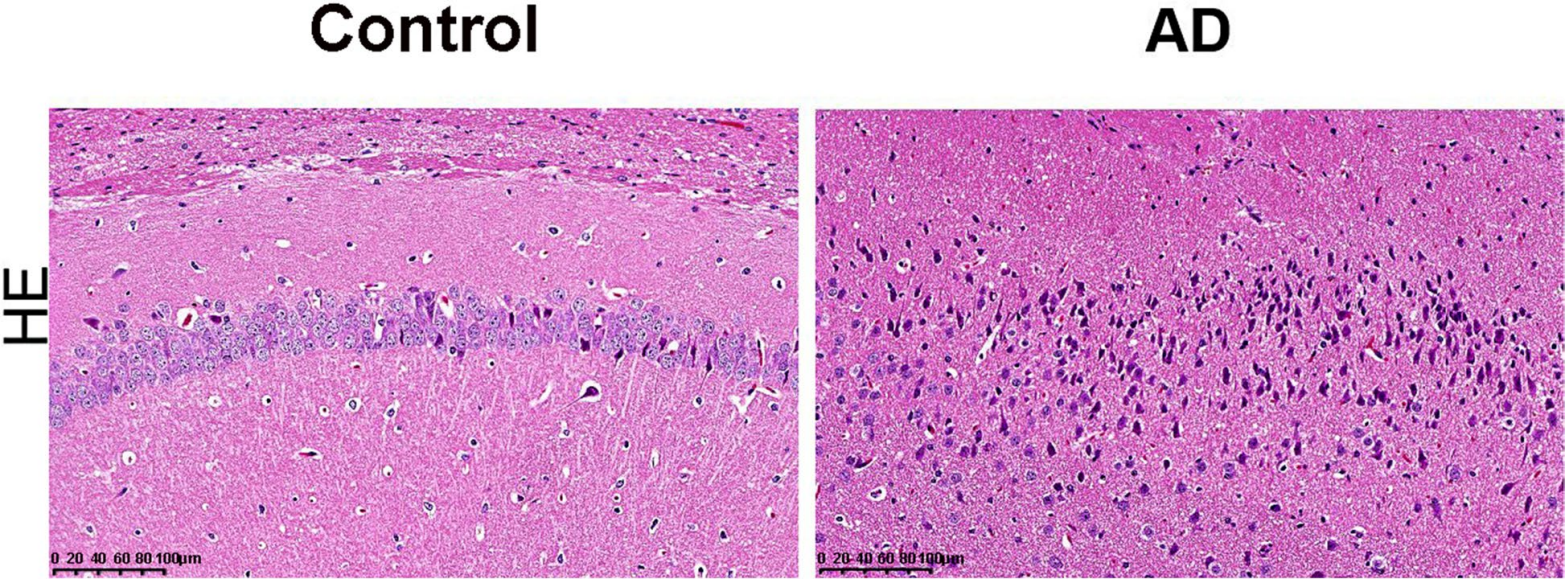
Hematoxylin-eosin staining results of brain tissues from AD mouse model (Magnification: 20×, scale: 100 μm)

The size, density, and arrangement of cortical nerve cells suggested the extent of neuron damage. In the control group, the cortical nerve cells of the mice brain tissue were densely organized, with full nucleoli and clear boundaries, which were in normal shape without pathological features. On the contrary, marked neuronal damage was observed in the mice of the model group. The cortical nerve cells of the model mice were overstained, loosely arranged, and their cytoplasm deformed and swollen, showing more lesions and irregular cell boundaries, which represented successful establishment of AD mice model (Fig. 7).

### RT-qPCR

**Fig. 8:**
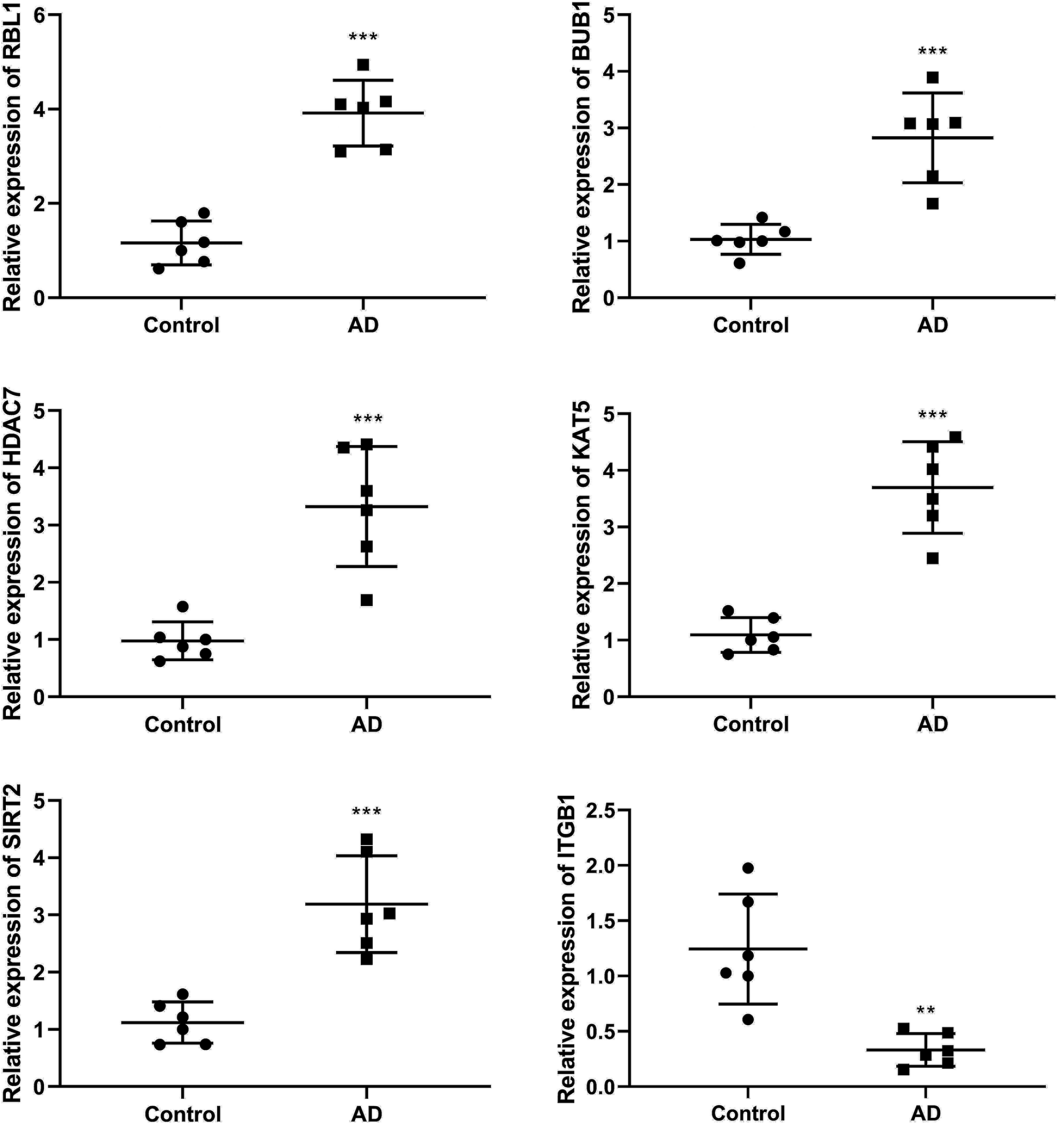
The mRNA expression expression levels of *RBL1, BUB1, HDAC7, KAT5, SIRT2, and ITGB1* detected by Real-time quantitative PCR. ^**^P < 0.01, ^***^P < 0.001 vs. control group

The results indicated that compared to the control group, the mRNA expression levels of *RBL1, BUB1, HDAC7, KAT5*, and *SIRT2* in the model group were significantly increased, while the mRNA expression level of ITGB1 was significantly decreased, consisting with the prediction of bioinformatics analysis (Fig. 8).

## Discussion

Currently, the treatment for AD can only alleviate its symptoms, which cannot suppress the progression of neurodegeneration and there is no effective treatment for people with AD [14]. Some oncogenes and suppressor genes have been found to be novel gene signatures for AD diagnosis [15, 16]. In this research, we identified 6 hub genes from PPI network based on 2 gene expression profiles related to AD. We then verified the abnormal expression of hub genes in vivo by constructing a mouse model of AD. Our findings not only provided new genetic diagnostic strategies, but also pointed the way to further study disease pathogenesis and therapeutic targets of AD.

*RBL1, BUB1, HDAC7, KAT5, SIRT2* and *ITGB1* have been identified as key genes in various diseases. By analyzing the clinical data of 4 glioma patients in Affymetrix chip, *RBL1* is isolated as a core gene affecting glioma patient survival and chemotherapy sensitivity [17]. *BUB1* is a gene signature that have a key effect on glioma stem cells after evaluating mRNA expression in glioma patient samples using The Cancer Genome Atlas and constructing DEGs co-expression networks of glioma samples by weighted gene co-expression network analysis [18]. Analysis of genomic data from 154 patients with glioblastoma multiforme has determined a strong correlation between *HDAC7* expression and patient outcomes: Subgroup of patients with overexpressed *HDAC7* has particularly poor clinical outcomes [19]. To investigating the role of Tip60 (now called *KAT5*) in human glioma malignant behavior, RT-qPCR analysis of fresh human brain tumor tissues from 55 patients shows that decreased *KAT5* expression is associated with advanced glioma, which is an important candidate gene for glioma [20]. Cerebrospinal fluid samples from patients with mild cognitive impairment caused by AD and a control group (i.e., patients with other neurological disorders) are analyzed using machine learning approaches, which identify *SIRT2* with high differential performance and can be used as a novel biomarker of neuroinflammation in AD [21]. After analyzing the datasets downloaded from GEO database to screen DEGs, performing GO and pathway enrichment analysis, constructing PPI, TF-target gene and miRNA-target gene networks, *ITGB1* is one of the hub genes that have been identified as possible targets for AD diagnosis and treatment [22]. In our research, after analyzing GSE5281 and GSE28146 datasets, *RBL1, BUB1, HDAC7, KAT5, SIRT2* and *ITGB1* were screened out with high predictive value, which might be important diagnostic markers for AD.

*RBL1* can inhibit the cell cycle and act as a disease suppressor [23–25]. In glioma, the expression level of *RBL1* is markedly down-regulated in glioma samples, playing a crucial role in glioma tumorigenesis [26]. *BUB1* is a mitotic kinase whose overexpression leads to aneuploidy, resulting in brain aging in health and disease [27, 28]. Compared with normal control tissues, *BUB1* expression is significantly up-regulated in glioblastoma multiforme samples [29]. *HDAC7* is a kind of histone deacetylases, and some histone deacetylases are associated with memory impairment and dementia [30–32]. *HDAC7* expression is up-regulated in malignant glioma, colorectal cancer, choroidal melanoma and other diseases [33–35]. *KAT5* acetylates and activates p53, which plays an important role in various cellular functions, associated with ageing-related diseases, including AD [36]. Transcriptomic analysis using the childhood cerebral cortex cell line as a neuronal model of novel coronavirus infection finds a decreased mRNA expression level of *KAT5* [37]. *SIRT2* is a sirtuin that is involved in aging, autophagy and inflammation, etc. [38, 39]. The expression level of *SIRT2* is up-regulated in brains of an insulin-deficient amyloid-β precursor protein transgenic mouse [40]. Additionally, the expression level of *ITGB1* in an orthotopic xenograft model of invasive glioblastoma is down-regulated [41]. In the brain tissue of a mouse model of AD that we constructed, the mRNA levels of *RBL1, BUB1, HDAC7, KAT5,* and *SIRT2* significantly increased, while the mRNA level of *ITGB1* significantly decreased. In conclusion, *RBL1, BUB1, HDAC7, KAT5, SIRT2*, and *ITGB1* were involved in AD, which were the crucial biomarkers for AD.

All in all, the identification of key hub gene of AD using bioinformatics techniques is the main focus in this study. Among the DEGs, 6 hub genes, *RBL1, BUB1, HDAC7, KAT5, SIRT2*, and *ITGB1* were acquired. Their abnormal expression was verified in vivo using AD mouse model. These hub genes have the potential to be novel treatment targets and biomarkers for AD patients. It is believed that our discovery would considerably advance knowledge of the underlying molecular mechanisms and causes of AD.

### Supplementary Information

**Additional file 1: Table S1.** The primer sequences.

**Additional file 2: Fig. S1.** Protein-protein interactions (PPI) network and key gene modules: Fig. A: PPI of DEGs. Fig. B: Hub genes identified using MCODE analysis. The lines between nodes represent the interactions between genes.

## Data Availability

The Gene expression profiles GSE5281 and GSE28146 analyzed during the current study are available in the Gene Expression Omnibus database at https://www.ncbi.nlm.nih.gov/geo/query/acc.cgi?acc=GSE28146, respectively.
