# Supplementary material for: Bioinformatic identification and experiment validation reveal 6 hub genes, promising diagnostic and therapeutic targets for Alzheimer’s disease
Source: BMC Med Genomics. 2024 Jan 2;17:6. doi: 10.1186/s12920-023-01775-6 (PMC10763315; doi:10.1186/s12920-023-01775-6)
Supplement: Supplementary file 1 — Additional file 1: Table S1. The primer sequences. [file 12920_2023_1775_MOESM1_ESM.docx]

Table S1 The primer sequences

| Name | Sequences（5’-3’） |
| --- | --- |
| GAPDH-F | GAGTCAACGGATTTGGTCGT |
| GAPDH-R | TTGATTTTGGAGGGATCTCG |
| RBL1-F | GGAGATTGGAACACCTCGAA |
| RBL1-R | AAGCTACAGGCGTGGTGACT |
| BUB1-F | TGCATTTTGAAGGTGCAGAG |
| BUB1-R | GCGAAAGTGAGGACAAGAGC |
| HDAC7-F | TGAAGAATGGCTTTGCTGTG |
| HDAC7-R | CACTGGGGTCCTGGTAGAAA |
| KAT5-F | CCAAGGAAAAGGAATCCACA |
| KAT5-R | TGCATGTGAAGCACAGATGA |
| SIRT2-F | AGCCAACCATCTGCCACTAC |
| SIRT2-R | CCAGCCCATCGTGTATTCTT |
| ITGB1-F | TGGACAATGTCACCTGGAAA |
| ITGB1-R | TGTGCCCACTGCTGACTTAG |
