# Supplementary material for: Bioinformatic identification and experiment validation reveal 6 hub genes, promising diagnostic and therapeutic targets for Alzheimer’s disease
Source: BMC Med Genomics. 2024 Jan 2;17:6. doi: 10.1186/s12920-023-01775-6 (PMC10763315; doi:10.1186/s12920-023-01775-6)

**Figure S1** Protein-protein interactions (PPI) network and key gene modules: Figure A: PPI of DEGs. Figure B: Hub genes identified using MCODE analysis. The lines between nodes represent the interactions between genes.


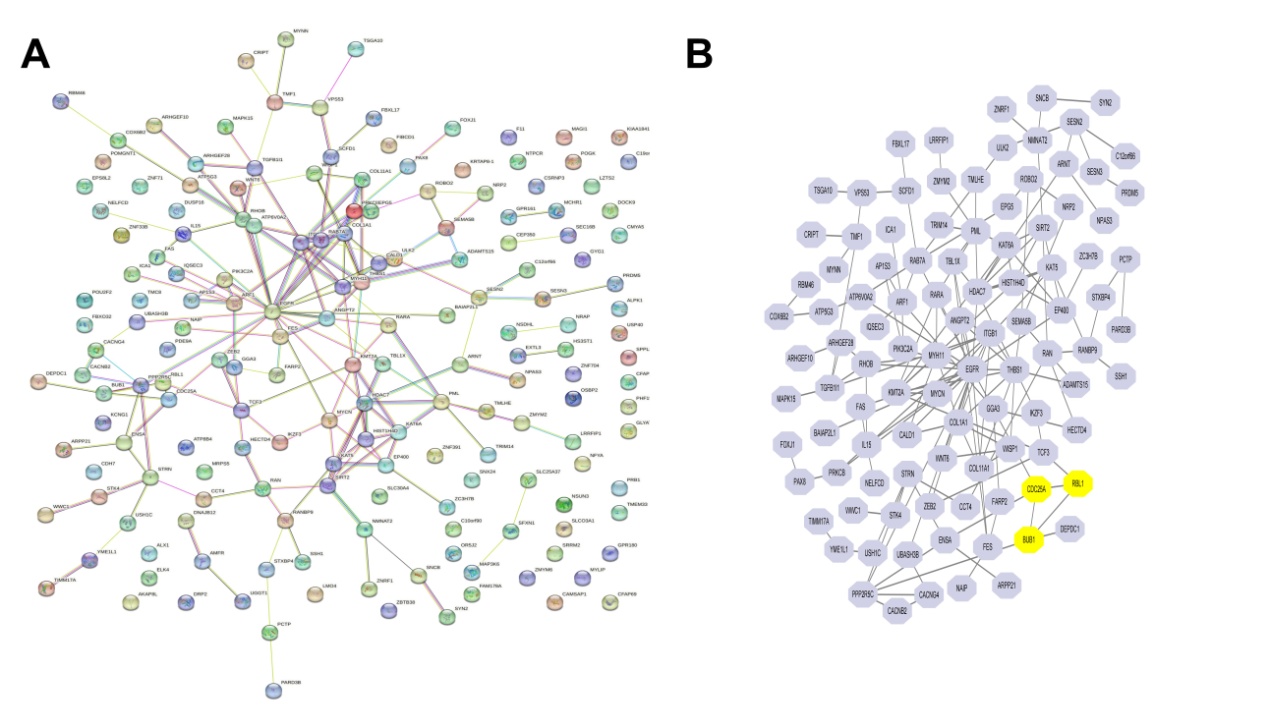

Supplement: Supplementary file 2 — Additional file 2: Fig. S1. Protein-protein interactions (PPI) network and key gene modules: Fig. A: PPI of DEGs. Fig. B: Hub genes identified using MCODE analysis. The lines between nodes represent the interactions between genes. [file 12920_2023_1775_MOESM2_ESM.docx]
